# Association between plaque characteristics and side-branch compromise in left main bifurcation lesions after a single-stent crossover technique: insights from an optical coherence tomography study

**DOI:** 10.3389/fcvm.2026.1760407

**Published:** 2026-03-06

**Authors:** Jincheng Han, Huai Yu, Maoen Xu, Tao Chen, Chao Fang, Xingtao Huang, Jinfeng Tan, Lijia Ma, Huimin Liu, Zhuozhong Wang, Guo Wei, Yanchao Liu, Haibo Jia, Bo Yu

**Affiliations:** 1 Department of Cardiology, The Second Affiliated Hospital of Harbin Medical University, Harbin, China; 2 The Key Laboratory of Myocardial Ischemia, Chinese Ministry of Education, Harbin, China

**Keywords:** left circumflex ostium compromise, left main bifurcation, optical coherence tomography, plaque characteristics, single-stent

## Abstract

**Background:**

The primary mechanism underlying side branch (SB) ostial compromise following main vessel (MV) stenting is the MV carina shift. However, the association between plaque characteristics or distribution and SB compromise remains unclear in patients with left main (LM) bifurcation lesions.

**Methods:**

A total of 123 patients with LM bifurcation lesions were included in the final analysis. Preprocedural optical coherence tomography (OCT) assessment of the LM-to-left anterior descending artery (LAD) segment was performed, and patients were treated with a single-stent crossover technique. Quantitative coronary angiography was performed to evaluate LM, LAD, and proximal left circumflex artery (LCX). Left circumflex artery ostium (LCX-OS) compromise was defined as residual stenosis > 50% after MV stenting.

**Results:**

LCX-OS compromise was observed in 33 patients in this study. Compared to the no compromise LCX-OS group, the compromise group had a higher frequency of plaque distribution in the LAD (90.9% vs. 65.6%, *p* < 0.001), more prominent calcified plaque characteristics (60.6% vs. 24.4%, *p* < 0.001), and a smaller LAD-LCX angle (81.2 ± 20.1 vs. 98.0 ± 25.7, *p* = 0.001). Adjusted multivariate logistic regression analysis revealed that plaques distributed in the proximal LAD (OR, 6.119; *p* = 0.011), calcified plaque (OR, 6.511; *p* = 0.001), and the LAD-LCX angle (OR, 0.966; *p* = 0.003) were independent predictors of LCX-OS compromise.

**Conclusions:**

Plaque in the proximal LAD, presence of calcified plaque, and a smaller LAD-LCX angle may contribute to LCX-OS compromise following MV stenting in patients with LM bifurcation lesions. OCT has emerged as a promising tool for preprocedural risk stratification in LM bifurcation percutaneous coronary intervention, helping to mitigate the risk of LCX-OS compromise.

## Introduction

Despite advances in interventional techniques and device technology, left main (LM) bifurcation lesions remain a clinical challenge for interventional cardiologists. Unlike non-LM coronary bifurcation lesions, the left circumflex artery (LCX), an LM side branch, is associated with greater clinical significance in terms of adverse outcomes. Furthermore, patients treated with a provisional single-stent technique demonstrated superior clinical outcomes compared with those treated with the planned two-stent strategy (1). A key risk associated with the provisional single-stent technique is LCX compromise after stent placement. Carina shift, primarily influenced by distal main vessel (MV) lumen expansion, is the primary mechanism of SB compromise after MV stent implantation in patients treated with a single-stent crossover stent (2). Optical coherence tomography (OCT) provides three-dimensional geometric information on plaque distribution and detailed insights into the pathophysiological and histological characteristics of lesions, thereby enabling its widespread application in the guidance and optimization of coronary interventions (3, 4). This study was designed to explore the role of OCT in evaluating the influence of MV lesion characteristics on LM bifurcation lesions treated with the single-stent crossover technique. Additionally, we aimed to investigate the correlation between MV lesion morphology and LCX-OS compromise.

## Materials and methods

### Patient population

**Figure 1:**
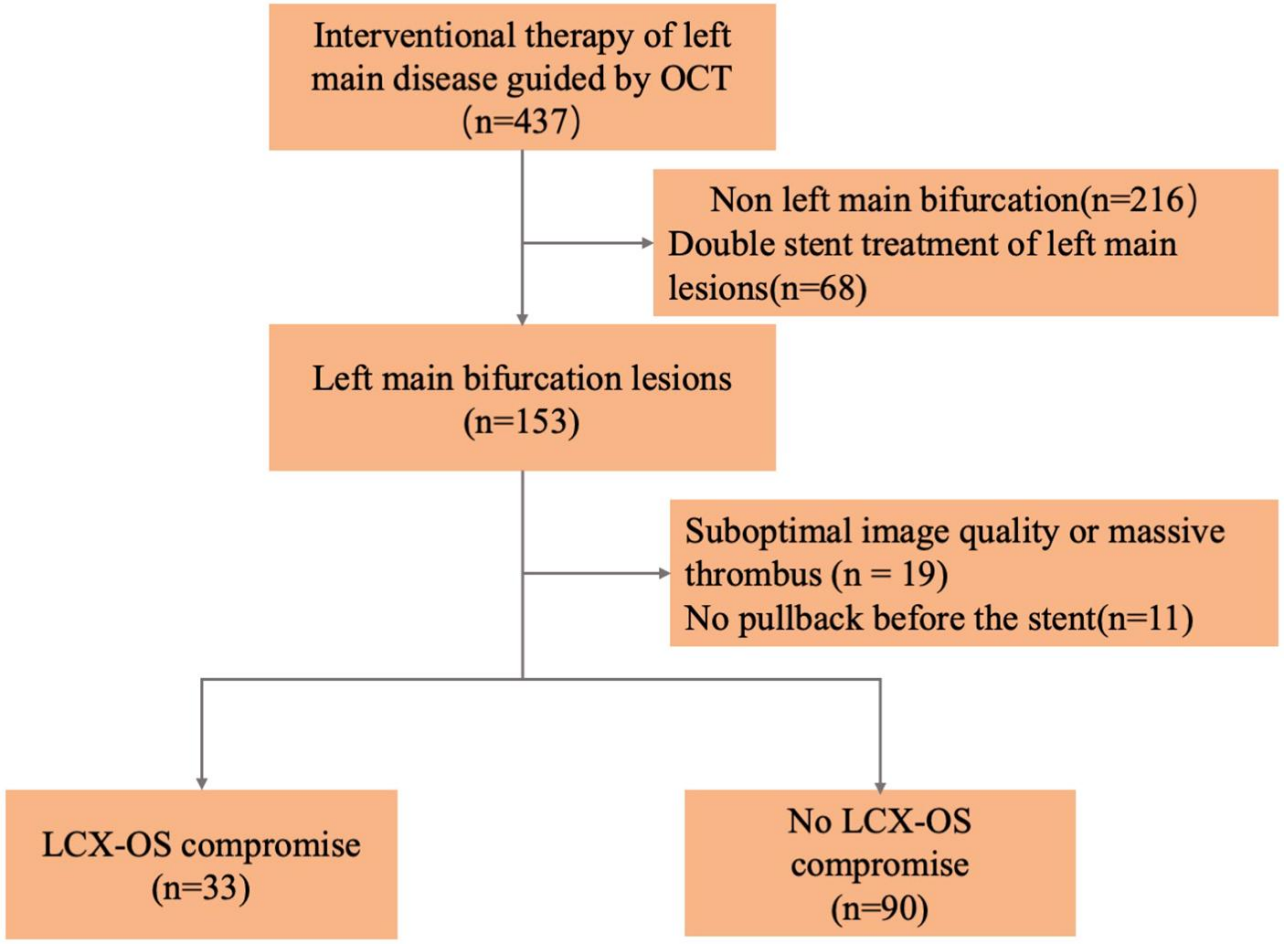
The flowchart of patient enrollment.

Initially, 437 patients with culprit LM lesions who underwent pre-interventional OCT examination (from the LM to LAD) at the Second Affiliated Hospital of Harbin Medical University (Harbin, China) were included in this study. The inclusion criteria were as follows: Patients with significant LM coronary artery disease (angiographic stenosis >50%), distal bifurcation lesions classified as Medina (1, 1, 0), and mild LCX disease (angiographic stenosis <50%). The exclusion criteria were as follows: Non-bifurcation lesions (*n* = 216), treatment with a two-stent strategy (*n* = 68), absence of pre-stenting OCT images (*n* = 11), poor image quality, or massive thrombus (*n* = 19). Finally, 123 patients with LM bifurcation lesions were included in the final analysis. A flowchart of the study is presented in Figure 1.

### Angiographic analysis

Quantitative coronary angiography (QCA) analysis was performed using a Cardiovascular Angiography Analysis System (version 5.10; Pie Medical Imaging B.V., Maastricht, the Netherlands). QCA parameters, including reference vessel diameter (RVD), minimal lumen diameter (MLD), degree of diameter stenosis (DS), and angle between LAD and LCX, were measured in end-diastolic frames. The detailed methodological procedures have been described in previous studies (5, 6). The Medina classification was used to characterize the location and distribution of the culprit bifurcation lesions (7). The 5-mm segments of LAD and LCX proximal to the carina were defined as the ostial LAD and ostial LCX, respectively. The LAD-LCX angle was measured before stent implantation. The confluence zone of the LAD and LCX was defined as the Polygon of Confluence (POC) (8). The distal LM segment above the POC was evaluated both pre- and post-stent implantation. LCX-OS compromise was defined as residual DS > 50% at the LCX-OS after main-vessel stent implantation and post-dilation.

### OCT image acquisition and analysis

OCT was performed using a commercially available C7-XR/ILUMIEN OCT system (Abbott Vascular, Santa Clara, California, United States). All OCT images were submitted to the Core Intravascular Imaging Laboratory of the Second Affiliated Hospital of Harbin Medical University, where they were analyzed by two independent investigators blinded to the patients' clinical and laboratory data using an off-line review workstation (Abbott Vascular). Any discrepancies in the analysis were resolved by consensus with a third senior reviewer.

Quantitative and qualitative analyses of lesions were performed before stent implantation in each of three segments: the ostial LAD, the POC, and the LM coronary artery above the POC. Specific criteria for evaluating plaque characteristics were applied, as previously described (9–11). Plaques were categorized into three types: lipid, fibrous, and calcified. Lipid plaques were defined as lesions with a signal-rich fibrous cap, a signal-poor region, and a diffuse border. For each lipid plaque, the minimum fibrous cap thickness (FCT) and maximum lipid arc were measured. The fibrous plaque was characterized as a homogeneous, highly backscattered region with low attenuation. Calcified plaques were identified as low-scattering regions with sharp borders; for each calcified plaque, the maximum calcified arc, calcium length, and calcium depth were quantified. Spotty calcium deposits were defined as those with a length <4 mm and a maximal arc <90°; deposits that did not meet these criteria were classified as large calcium deposits.

### Statistical analysis

Statistical analyses were conducted by an independent statistician using R software (version 4.1.2). Data distribution was evaluated using the Kolmogorov–Smirnov test. Continuous variables were compared using the independent-samples Student's *t*-test or Mann–Whitney *U*-test, with mean ± standard deviation for normally distributed data and median (interquartile range) for non-normally distributed data. Categorical variables were compared using the chi-square test and are presented as counts (proportions). Fisher's exact test was applied when the expected frequency of any cell was <5. The associations between plaque characteristics and distribution were analyzed using a logistic regression model. Variables with *p*-values <0.2 in the univariate logistic regression analysis were included in the multivariable model using stepwise selection. A two-sided *p*-value <0.05 was considered statistically significant.

## Results

### Clinical characteristics

This study included 123 patients with culprit LM bifurcation lesions. After stent implantation from LM to LAD, 33 patients developed LCX-OS compromise, while 90 did not. The baseline clinical and procedural characteristics are presented in Table 1. No statistically significant differences in the baseline clinical characteristics were observed between the two groups (LCX-OS compromise vs. no LCX-OS compromise).

**Table 1:** Baseline clinical and procedural characteristics.

Variables	No LCX-OS compromise *n* = 90	LCX-OS compromise *n* = 33	*t*/χ^2^	*P*
Age, mean ± SD	63.6 ± 8.8	62.3 ± 11.0	0.671	0.504
Male, *n* (%)	67 (74.4)	24 (72.7)	0.037	0.847
Hypertension, *n* (%)	43 (47.8)	16 (48.5)	0.005	0.945
Diabetes mellitus, *n* (%)	32 (35.6)	9 (27.3)	0.745	0.388
Smokers, *n* (%)	32 (35.6)	11 (33.3)	0.052	0.819
Alcohol users, *n* (%)	14 (15.6)	2 (6.1)	1.176	0.278^a^
Total cholesterol (mmol/L)	4.2 ± 1.0	4.1 ± 0.8	0.388	0.699
Low-density lipoprotein (mmol/L)	2.5 ± 0.9	2.4 ± 0.8	0.735	0.464
Triglyceride (mmol/L)	1.7 ± 0.9	1.5 ± 0.5	0.640	0.523
Medina classification				
1,1,0	41 (45.6)	17 (51.5)	0.785	0.675
1,0,0	20 (22.2)	5 (15.2)		
0,1,0	29 (32.2)	11 (33.3)		
Stent diameter, mm	3.5 ± 0.4	3.5 ± 0.4	0.077	0.939
Maximal balloon pressure	19.6 ± 3.3	19.1 ± 3.7	0.715	0.476
Maximal balloon size, mm	3.7 ± 0.4	3.7 ± 0.4	−0.154	0.878
Type of stent				
Zotarolimus-eluting stent	2 (2.2)	1 (3.0)	0.266	0.872^a^
Everolimus-eluting stent	16 (17.8)	7 (21.2)		
Rapamycin-eluting stent	72 (80.0)	25 (75.8)		

“a” denotes cells with an expected frequency < 5; results were obtained using the chi-square test with continuity correction.

### Angiographic and procedural findings

Pre-stent implantation QCA results, including RVD, MLD, and DS, in the distal LM coronary artery and proximal LAD were balanced between the LCX-OS compromise and no LCX-OS compromise groups (Table 2). No significant difference was observed in the pre-stent MLD (2.2 ± 0.5 mm vs. 2.2 ± 0.5 mm, *p* = 0.587) or DS (15.4 ± 6.7% vs. 17.5 ± 8.5%, *p* = 0.155) of the LCX between the two groups. However, the LCX-OS compromise group had a significantly smaller LAD-LCX angle than the no LCX-OS compromise group (81.2 ± 20.1° vs. 98.0 ± 25.7°, *p* = 0.001). After single-stent crossover technique, the LCX-OS compromise group had a significantly smaller MLD (1.3 ± 0.2 mm vs. 2.1 ± 0.5 mm, *p* < 0.001) and a significantly higher DS (51.9 ± 2.1% vs. 20.1 ± 9.6%, *p* < 0.001) than the no LCX-OS compromise group.

**Table 2:** Quantitative coronary angiographic data.

Variables	No LCX-OS compromise *n* = 90	LCX-OS compromise *n* = 33	*t*	*P*
Pre-stent				
Distal LM				
RD (mm)	3.8 ± 0.5	3.9 ± 0.5	−0.870	0.386
MLD (mm)	2.3 ± 1.1	2.3 ± 0.9	−0.099	0.921
DS (%)	40.1 ± 25.3	40.5 ± 22.0	−0.080	0.937
Proximal LAD				
RD (mm)	2.8 ± 0.5	3.0 ± 0.4	−1.552	0.123
MLD (mm)	1.4 ± 0.7	1.4 ± 0.8	0.209	0.835
DS (%)	49.9 ± 24.5	54.8 ± 23.7	−1.000	0.319
Proximal LCX				
RD (mm)	2.6 ± 0.6	2.7 ± 0.5	−0.063	0.950
MLD (mm)	2.2 ± 0.5	2.2 ± 0.5	0.545	0.587
DS (%)	15.4 ± 6.7	17.5 ± 8.5	−1.432	0.155
Angle: LAD-LCX	98.0 ± 25.7	81.2 ± 20.1	3.378	**0** **.** **001**
Post-stent				
Distal LM				
RD (mm)	4.0 ± 0.5	4.1 ± 0.3	−1.301	0.196
MLD (mm)	3.4 ± 0.4	3.5 ± 0.4	−0.468	0.640
DS (%)	13.2 ± 8.5	14.2 ± 9.5	−0.582	0.562
Proximal LAD				
RD (mm)	3.3 ± 0.4	3.4 ± 0.5	−0.738	0.462
MLD (mm)	3.0 ± 0.4	3.0 ± 0.5	0.006	0.996
DS (%)	8.7 ± 7.3	10.4 ± 7.5	−1.121	0.264
Proximal LCX				
RD (mm)	2.6 ± 0.5	2.7 ± 0.5	−0.774	0.440
MLD (mm)	2.1 ± 0.5	1.3 ± 0.2	9.370	**<0** **.** **001**
DS (%)	20.1 ± 9.6	51.9 ± 2.1	−18.783	**<0** **.** **001**

RD, reference diameter; MLD, minimal luminal diameter; DS, diameter stenosis, LM, left main, LAD, left anterior descending artery, LCX, left circumﬂex.

Bold values indicate statistical significance (*P* < 0.05).

### OCT findings

**Figure 2:**
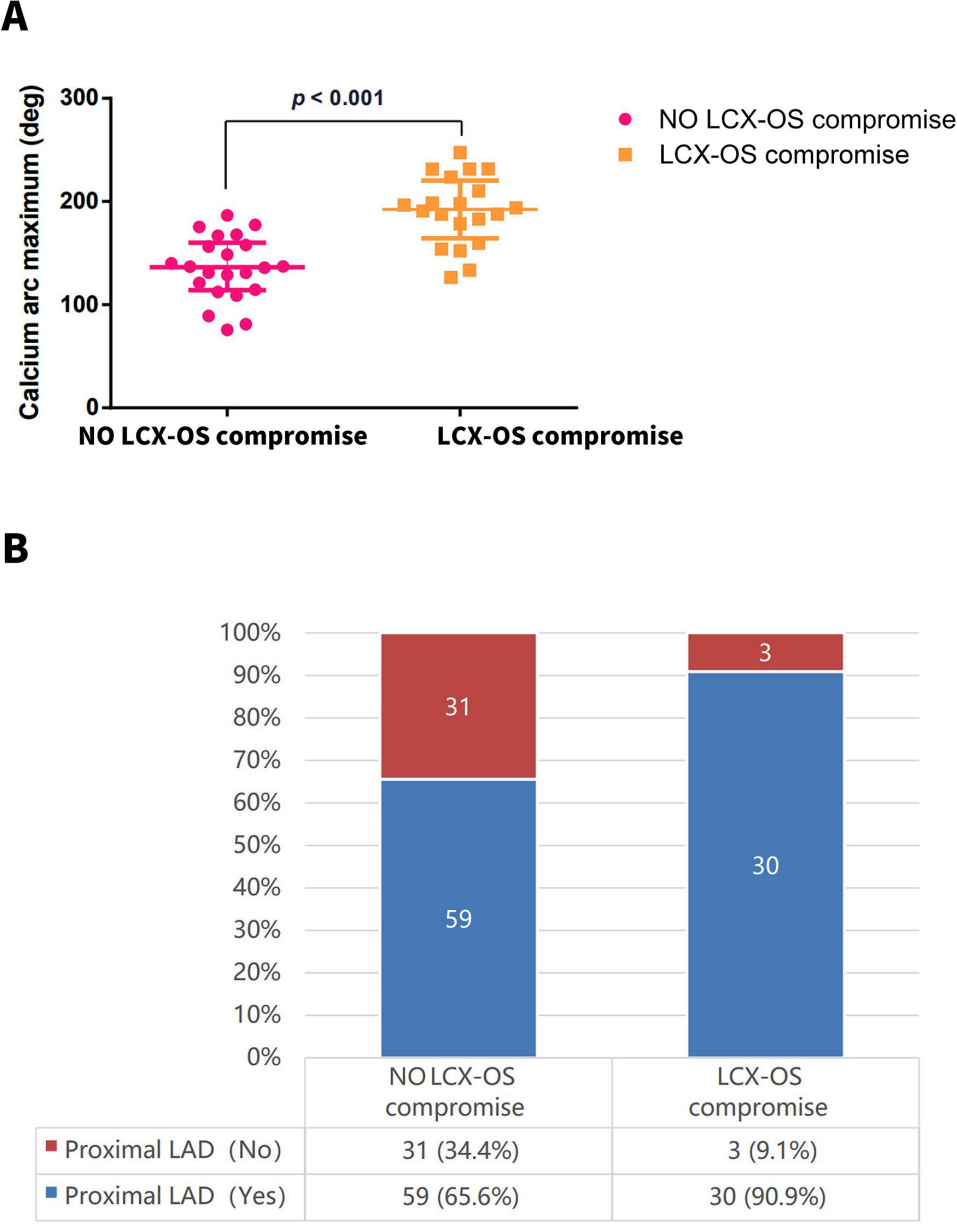
**(A)** the calcification arc was significantly larger in the LCX-OS group than in the no LCX-OS compromise group. **(B)** The LCX-OS compromise group had a significantly higher frequency of plaque distribution in the proximal LAD than the no LCX-OS compromise group. LCX-OS, left circumflex artery ostium; LAD, left anterior descending artery.

The OCT findings of the LCX-OS compromise and no LCX-OS compromise groups are compared in Table 3. The LCX-OS compromise group had a larger minimal lumen area (MLA) in the target lesion than the no LCX-OS compromise group (2.0 ± 0.6 mm^2^ vs. 1.7 ± 0.7 mm^2^, *p* = 0.018), but the area stenosis was similar between the two groups (80.4% ± 7.9% vs. 78.7% ± 6.2%, *p* = 0.271). Compensatory vascular dilation in the compromise group may account for the preserved MLA, with the relative plaque proportion remaining unchanged despite luminal enlargement and comparable to the no compromise group. Additionally, discrepancies in plaque distribution may be an additional factor related to this result. The proportion of patients with calcified lesions was significantly higher in the LCX-OS compromise group than in the no-LCX-OS compromise group (60.6% vs. 24.4%, *p* < 0.001). Moreover, among patients with calcified lesions, the LCX-OS compromise group exhibited a significantly larger maximum calcium arc (190.8 ± 30.4° vs. 135.5 ± 30.7°, *p* < 0.001) and longer calcium length (10.2 ± 7.6 mm vs. 5.8 ± 2.7 mm, *p* = 0.014) than the no LCX-OS compromise group (Figure 2). Furthermore, the LCX-OS compromise group had a significantly higher frequency of plaque distribution in the proximal LAD than the no LCX-OS compromise group (90.9% vs. 65.6%, *p* = 0.005; Figure 2). Plaque distribution at the distal LM and POC showed no significant difference between the two groups. However, the distribution features suggest indirectly that plaques were diffusely distributed in both groups, mostly extending from the distal LM to the POC or from the proximal LAD to the POC.

**Table 3:** Pre-stent implantation OCT findings from the LAD to the LM.

Variables	No LCX-OS compromise *n* = 90	LCX-OS compromise *n* = 33	*t*	*P*
MLA, mm^2^	1.7 ± 0.7	2.0 ± 0.6	−3.495	**0** **.** **018**
Mean RVA, mm^2^	9.1 ± 2.1	9.9 ± 2.3	−2.697	0.092
Stenosis area, %	80.4 ± 7.9	78.7 ± 6.2	1.106	0.271
Plaque type				
Lipid plaque, *n* (%)	52 (57.8)	14 (42.4)	2.289	0.130
Lipid length, mm	7.4 ± 3.1	6.7 ± 2.5	0.667	0.507
Lipid arc (deg)	193.6 ± 48.1	185.1 ± 65.2	0.444	0.659
Minimal FCT, μm	97.2 ± 41.7	70.0 ± 27.8	1.777	0.081
Fibrous *n* (%)	68 (75.6)	27 (81.8)	0.539	0.463
Calcification plaque, *n* (%)	22 (24.4)	20 (60.6)	14.042	**<0** **.** **001**
Calcium arc maximum (deg)	135.5 ± 30.7	190.8 ± 33.4	−5.591	**<0** **.** **001**
Calcium length, mm	5.8 ± 2.7	10.2 ± 7.6	−2.561	0.014
Calcium depth, mm	0.6 ± 0.2	0.6 ± 0.3	−0.295	0.769
Spotty calcification, *n* (%)	5 (5.6)	5 (15.2)	2.977	0.084
Plaque distribution				
Distal LM	53 (58.9)	14 (42.4)	2.639	0.104
POC	78 (86.7)	26 (78.8)	1.148	0.284
Proximal LAD	59 (65.6)	30 (90.9)	7.760	**0** **.** **005**

MLA, minimal luminal area, RVA, reference vessel area, POC, polygon of confluence, LM, left main, LAD, left anterior descending artery.

Bold values indicate statistical significance (*P* < 0.05).

**Figure 3:**
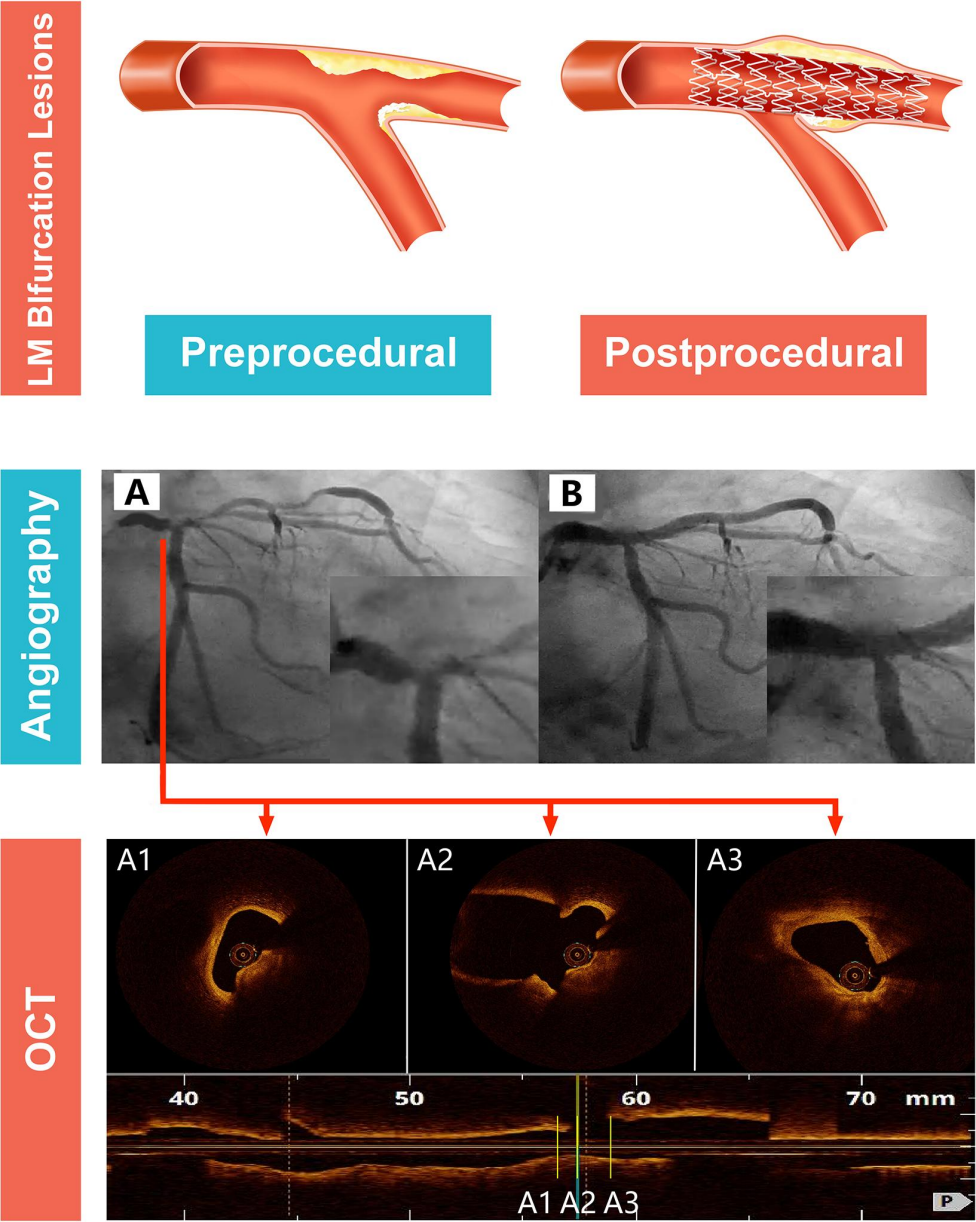
Angiographic findings before single-stent implantation exhibited no stenosis at LCX-OS; however, a significant LCX-OS compromise was observed after stenting. OCT revealed that the lesion was predominantly in the proximal LAD and partially extended into the distal LM, with a calcified plaque as its key pathological feature. LM, left main; OCT, optical coherence tomography.

Other plaque characteristics, including lipid content, lipid arc, minimal FCT, fibrous content, and spotty calcification, were similar between the two groups. LCX-OS and no LCX-OS compromise groups did not differ significantly in the frequency of fibrous or lipid components. Representative examples of angiographic and OCT appearances of LCX-OS compromise are presented in Figure 3.

### Multivariate logistic regression analysis of LCX-OS compromise

The OCT features with statistical significance in the univariate analysis (MLA, Angle: LAD-LCX, Calcification, and Proximal LAD) were further incorporated into the multivariate logistic regression model to evaluate the independent effects of each feature on grouping; after stepwise selection, only the latter three features were included in the optimal logistic regression model. In the multivariable logistic regression analysis, Model 1, including only clinically relevant factors that were statistically significant in the univariate analysis (LAD-LCX, Calcification, and Proximal LAD), revealed that three factors were independently associated with LCX-OS compromise after single-stent crossover implantation from the LM coronary artery to the LAD: LAD-LCX angle [odds ratio (OR), 0.968; 95% confidence interval (CI): 0.947–0.989; *p* = 0.003], presence of calcified lesions (OR, 5.356; 95% CI: 2.088–13.734; *p* < 0.001), and plaque distribution in the proximal LAD (OR, 5.346; 95% CI: 1.374–20.793; *p* = 0.016). Models 2 (adjusted for baseline factors: age, gender, history of hypertension, history of diabetes mellitus, smoking history, and alcohol consumption history) and 3 (adjusted for all factors in Model 2 plus stent-related variables: stent diameter, maximal balloon pressure, and maximal balloon size) consistently confirmed that the plaque distribution in the proximal LAD (OR, 6.119; *p* = 0.011), presence of calcified lesions (OR, 6.511; *p* = 0.001), and the LAD-LCX angle (OR, 0.966; *p* = 0.003), remained independent predictors of LCX-OS compromise (Table 4).

**Table 4:** Multivariate logistic regression analysis predictors for LCX-OS compromised.

Variables	Multivariate analysis (Model1)			Multivariate analysis (Model2)			Multivariate analysis (Model2)		
	OR	95% CI	*P*-value	OR	95% CI	*P*-value	OR	95% CI	*P*-value
Angle: LAD-LCX	0.968	0.947–0.989	**0** **.** **003**	0.967	0.946–0.989	**0**.**003**	0.966	0.945–0.988	**0** **.** **003**
Calcification plaque (Yes vs. No)	5.356	2.088–13.734	**<0** **.** **001**	5.496	2.012–15.017	**0**.**001**	6.511	2.246–18.877	**0** **.** **001**
Proximal LAD (Yes vs. No)	5.346	1.374–20.793	**0** **.** **016**	5.406	1.354–21.586	**0**.**017**	6.119	1.503–24.902	**0** **.** **011**
Age	–	–	–	0.993	0.945–1.044	0.781	0.998	0.948–1.050	0.933
Sex(Famale vs. Male)	–	–	–	0.792	0.243–2.579	0.699	0.856	0.240–3.049	0.810
Hypertension (Yes vs. No)	–	–	–	0.822	0.305–2.218	0.698	0.793	0.289–2.181	0.654
Diabetes mellitus (Yes vs. No)	–	–	–	0.623	0.209–1.857	0.396	0.602	0.200–1.807	0.366
Smokers (Yes vs. No)	–	–	–	0.812	0.248–2.650	0.729	0.907	0.272–3.019	0.873
Alcohol users (Yes vs. No)	–	–	–	0.382	0.062–2.358	0.300	0.248	0.034–1.804	0.169
Stent diameter	–	–	–	–	–	–	1.652	0.185–14.795	0.653
Maximal balloon pressure	–	–	–	–	–	–	0.992	0.863–1.140	0.911
Maximal balloon size	–	–	–	–	–	–	1.964	0.269–14.367	0.506

Model 1: Included only clinically relevant factors that were statistically significant in the univariate analysis.

Model 2: Adjusted for baseline factors, including age, sex, history of hypertension, history of diabetes mellitus, smoking history, and alcohol consumption history.

Model 3: Adjusted for all factors in Model 2 plus stent-related variables, namely stent diameter, maximal balloon pressure, and maximal balloon size.

## Discussion

In this study, we used pre-intervention OCT imaging of the distal LM bifurcation to assess plaque characteristics and distribution in the distal LM and explore their relationship with LCX-OS compromise after single-stent crossover technique. The primary findings of this study were as follows: (a) in the LCX-OS compromise group, both the proportion of calcified lesions and the arc of calcification were significantly higher than those in the no LCX-OS compromise group. (b) Plaques located in the proximal LAD were significantly more likely to be associated with LCX-OS compromise after the single-stent crossover technique for LM bifurcation lesions. (c) A smaller angle between LAD and LCX was associated with a higher risk of left LCX-OS compromise after single-stent crossover technique.

LM coronary artery disease imperils a large myocardial territory and elevates the risk of major adverse cardiovascular events (MACE). A review recommends that the provisional single-stent approach remains the most common treatment strategy for percutaneous coronary intervention in LM coronary artery disease, and invasive imaging and stent optimization techniques are key to achieving acute and long-term clinical success (12). The European Bifurcation Club (EBC) LM trial (a randomized, investigator-initiated, open-label, multicenter, parallel-group trial) demonstrated no significant difference in the incidence of MACE between the stepwise provisional stent strategy and the systematic dual-stent strategy at three years following percutaneous coronary intervention for LM bifurcation coronary artery disease. The stepwise provisional strategy should remain the default strategy for distal LM bifurcation intervention (13). A recent study demonstrated that clinical outcomes were significantly worse in patients with LM true bifurcation lesions compared to those with non-LM true bifurcation lesions (14). However, LCX-OS compromise following single-stent implantation for LM bifurcation lesions remains a clinical challenge that we need to address.

Ostial stenosis or occlusion of the SB arises from a combination of carinal and plaque shifts. Carinal shift occurs when the MV stent is oversized relative to the distal MV, leading to carina displacement into the SB. This phenomenon is more pronounced in SB with a high angle and small caliber, leading to thinning of the carinal segment, which is visualized as an “eyebrow sign” on intravascular imaging (15, 16). Carinal shift, a key mechanism underlying SB ostial compromise, has been supported by prior intravascular ultrasound (IVUS) evidence, exhibiting that 85% of the loss in SB ostial lumen volume following MV stenting was attributable to this shift (2).

The results of this study demonstrated that the group with LCX-OS compromise had a higher proportion of calcified lesions and a larger calcification arc. Furthermore, calcified plaques were independently associated with LCX-OS compromise after single-stent crossover technique. This phenomenon is attributed to the tendency of the expanded MV stent to displace the carina toward the LCX-OS after single-stent implantation for LM bifurcation lesions due to calcified lesions. This effect was particularly pronounced after post-dilation of the MV. Therefore, adequate identification of lesion characteristics before stent implantation, coupled with appropriate management of severe calcified lesions (rotational atherectomy or intravascular lithotripsy), may reduce the risk of LCX-OS compromise following single-stent implantation. In clinical practice, OCT's superior resolution underpins its core applications in bifurcation lesions. Preoperatively, it provides precise characterization of plaque composition (lipid, fibrous, calcified) and morphology, along with measurements of lesion length, vessel diameter, and bifurcation geometry—critical for formulating personalized percutaneous coronary intervention strategies. A study indicated that the presence of a bifurcating target vessel with moderate-to-severe calcification is associated with a higher risk of adverse outcomes than either attribute alone (17). Ours findings highlight that calcified lesions and their related characteristics (larger calcification arc) play a crucial role in mediating LCX-OS compromise after single-stent implantation, which is a common clinical challenge in managing LM bifurcation lesions.

Angiography fails to accurately localize and characterize plaques at the LM bifurcation lesion site. An IVUS study demonstrated that the carinal of LM bifurcation is rarely involved by plaque, with the disease predominantly distributed diffusely from the distal LM coronary artery to the proximal LAD (18). Findings from another study demonstrated that OCT imaging identified more diffuse lesions at the distal LM coronary artery than angiography, with these lesions predominantly distributed contralateral to the LCX-OS, extending from the distal LM to the proximal LAD (19). In this study, the proportion of patients with plaques located in the proximal LAD was significantly higher in the LCX-OS compromise group than in the no LCX-OS compromise group. Additionally, plaque distribution in the proximal LAD remained an independent predictor of LCX-OS compromise. Plaque displacement in the proximal LAD and expansion of stent struts toward the carina following single-stent implantation may represent the primary mechanism underlying LCX-OS compromise. Previous studies have reported that the mechanisms underlying lumen enlargement following stenting include significant axial redistribution of plaque from the lesion to the reference segments, vessel expansion, and plaque embolization or compression (20, 21).

Geometric alterations following stent placement in the MV from the distal LM to the LAD can cause acute compromise of the LCX-OS, with the primary mechanism being carina shift, which is associated with a narrow angle between LAD and LCX (22). Kang et al. (23) demonstrated that crossover stenting from the LM coronary artery to the LAD resulted in a >10% reduction in MLA at the LCX-OS, with a narrow LAD-LCX angle being associated with carina shift. Plaque redistribution, superimposed on geometric alterations, contributed to luminal loss at the LCX-OS. Consistent with these findings, this study revealed that a smaller LAD-LCX angle was associated with a higher risk of LCX-OS compromise following single-stent implantation. This may be attributed to the fact that a smaller LAD-LCX angle is associated with a more significant carina shift toward the LCX-OS following stent implantation. Nevertheless, despite the high stability exhibited by the adjusted multivariate logistic regression analysis, the limited number of LCX-OS compromise events introduces a potential risk of overfitting.

Percutaneous coronary intervention for LM bifurcation disease is a challenging procedure in interventional cardiology. When evaluating these lesions, numerous critical factors warrant consideration, including the lesion location, branch ostial involvement, lesion characteristics, bifurcation angle, and challenges associated with local drug delivery at the LCX-OS. Consequently, EBC has demonstrated that several protective strategies can prevent SB closure or mitigate compromise after MV stenting (24). IVUS and OCT are increasingly recognized as the gold standard for percutaneous coronary intervention in LM coronary bifurcation lesions, with accumulating evidence supporting superior outcomes over angiography alone (25, 26). Previous studies have demonstrated that SB compromise in general bifurcation lesions can be predicted through the OCT imaging features of the MV (27, 28). In contrast, the present study focuses on investigating the feasibility of using OCT to predict the risk of LCX-OS compromise during interventional treatment for LM bifurcation lesions.

Currently, OCT is the highest-resolution intracoronary imaging tool in clinical use. It enables clear visualization of plaque distribution, composition, and vascular bifurcation geometry, thereby exerting significant value in guiding PCI for bifurcation lesions. In particular, identifying the potential occurrence of carina shift and plaque shift prior to the application of single-stent technique in LM bifurcations holds significant guiding significance for preventing the involvement of LCX-OS. Previous study has reported that lesions with lipid-rich plaques in the MV at bifurcation sites are prone to plaque shift after PCI, which is associated with SB compromise (29). Based on this study's results, plaques in the proximal LAD may shift after PCI, causing LCX-OS compromise. We may consider using cutting balloons or non-slip element balloons for adequate lesion and provisional dual-stent strategies preconditioning to avoid LCX-OS compromise caused by plaque shift. In contrast, a smaller LAD-LCX angle and rigid calcified plaques may be more likely to cause carina shift. Of course, this inference requires further research to be validated. And, the jailed balloon technique can be used to prevent this situation from occurring. Accordingly, these findings confirm that preprocedural OCT enables the accurate identification of lesion characteristics in LM bifurcation disease before single-stent implantation. Subsequent lesion preparation tailored to OCT findings and the selection of an appropriate interventional strategy may prevent LCX-OS compromise following the procedure.

### Study limitations

This study has several limitations. First, this study adopted a retrospective design and selected a cohort of patients who underwent single-stent crossover implantation from the LM coronary artery to the LAD. This retrospective nature and cohort selection may introduce selection bias, potentially affecting the generalizability of our findings regarding the factors associated with LCX-OS compromise. Second, the sample size of this cohort was relatively small, which may limit the statistical power to detect subtle associations between the predictors and LCX-OS compromise. Third, the present study is the absence of functional and clinical endpoint assessments for the LCX-OS after stent implantation in the LAD to LM. In the future, additional randomized controlled trials are needed to provide more data and further validate the value of OCT in the interventional treatment of LM bifurcation lesions.

## Conclusions

Coronary calcification characteristics, proximal LAD plaque distribution, and a smaller LAD-LCX angle are associated with LCX-OS compromise in patients with LM bifurcation lesions treated with the single-stent crossover technique. Furthermore, OCT has emerged as a promising tool for preprocedural risk stratification during percutaneous coronary intervention for LM bifurcation lesions, potentially mitigating the risk of LCX-OS compromise.

## Data Availability

The raw data supporting the conclusions of this article will be made available by the authors, without undue reservation.
